# Individualized multi-modal MRI biomarkers predict 1-year clinical outcome in first-episode drug-naïve schizophrenia patients

**DOI:** 10.3389/fpsyt.2024.1448145

**Published:** 2024-09-13

**Authors:** Aoxiang Zhang, Chenyang Yao, Qian Zhang, Ziyuan Zhao, Jiao Qu, Su Lui, Youjin Zhao, Qiyong Gong

**Affiliations:** ^1^ Department of Radiology, West China Hospital of Sichuan University, Chengdu, China; ^2^ Research Unit of Psychoradiology, Chinese Academy of Medical Sciences, Chengdu, China; ^3^ Department of Radiology and Nuclear Medicine, Xuanwu Hospital Capital Medical University, Beijing, China; ^4^ Department of Radiology, and Functional and Molecular Imaging Key Laboratory of Sichuan Province, West China Hospital of Sichuan University, Chengdu, China; ^5^ Department of Radiology, West China Xiamen Hospital of Sichuan University, Xiamen, Fujian, China

**Keywords:** antipsychotic medication, individualized imaging biomarker, machine learning, schizophrenia, treatment-resistant

## Abstract

**Background:**

Antipsychotic medications offer limited long-term benefit to about 30% of patients with schizophrenia. We aimed to explore the individual-specific imaging markers to predict 1-year treatment response of schizophrenia.

**Methods:**

Structural morphology and functional topological features related to treatment response were identified using an individualized parcellation analysis in conjunction with machine learning (ML). We performed dimensionality reductions using the Pearson correlation coefficient and three feature selection analyses and classifications using 10 ML classifiers. The results were assessed through a 5-fold cross-validation (training and validation cohorts, n = 51) and validated using the external test cohort (n = 17).

**Results:**

ML algorithms based on individual-specific brain network proved more effective than those based on group-level brain network in predicting outcomes. The most predictive features based on individual-specific parcellation involved the GMV of the default network and the degree of the control, limbic, and default networks. The AUCs for the training, validation, and test cohorts were 0.947, 0.939, and 0.883, respectively. Additionally, the prediction performance of the models constructed by the different feature selection methods and classifiers showed no significant differences.

**Conclusion:**

Our study highlighted the potential of individual-specific network parcellation in treatment resistant schizophrenia prediction and underscored the crucial role of feature attributes in predictive model accuracy.

## 1 Introduction

Schizophrenia is a complex and heterogeneous behavioral and cognitive syndrome that appears to stem from a combination of genetic and environmental factors, resulting in impaired brain development (1). Treatment resistant schizophrenia (TRS) is a condition wherein a significant proportion of individuals with schizophrenia continue to experience symptoms and poor outcomes despite treatment (2). Antipsychotic medications provide limited longer-term benefits to approximately 30% of schizophrenia patients (1–3). Therefore, early prediction of TRS and the tailored administration of appropriate interventions can alleviate the burden on affected individuals and avoid more serious consequences (4), such as extreme despair and increased risk of suicide (5).

Neuroimaging has been used to investigate brain features of TRS and is expected to provide biomarkers closer to biology for predicting TRS than clinical indicators, including poor premorbid social functioning (2), early age of onset (6, 7), and history of drug or alcohol abuse (8). Our previous study has demonstrated the ability to identify TRS in patients with schizophrenia using structural and functional neuroimages before treatment (9). Previous structural MRI studies have reported that TRS patients exhibit widespread gray matter volume (GMV) reduction compared with both of healthy controls and treatment-responsive group in many brain regions, such as the frontal, temporal, postcentral, occipital cortices, and hippocampus (10–12). Graph theory analysis (GTA) offers a method for simplifying complicated networks into clearer representations, facilitating the assessment of brain organization. A study has shown diminished global efficiency and heightened local efficiency in TRS (13). Additionally, an increased clustering coefficient was associated with the improvement of negative symptom in schizophrenia (14). Despite the large number of structural and functional magnetic resonance imaging (MRI) findings on TRS in previous studies (9–14), predicting the course of an individual’s disease, especially the likelihood of treatment response, is very challenging (4). The development of neuroimaging markers has proved elusive due to the complex, distributed, and subtle variations that depend on an individual’s unique clinical characteristics. Traditional analytical methods, which provide average estimates at the group level, have proved insufficient to detect such variations and deal with inter-individual heterogeneity (15–17).

In order to address this challenge, researchers have begun using an alternative analysis method known as machine learning (ML). It aims to construct models that support individual predictions, thus shifting from the study of univariate statistical group differences towards multivariate complex brain patterns of individual patients (15). Several studies have applied ML techniques and MRI to identify biomarkers of treatment response in schizophrenia with varying degree of success (18–22). Prediction accuracies typically range between 60 and 90%. One study used random forest models derived from thalamic shape information to predict TRS with 75% accuracy (22). Functional connectivity of the superior temporal cortex was informative in predicting response to antipsychotics (83% accuracy) using support vector machine (SVM) algorithm (20). Despite the excellent performance of the predictive models in disparate studies, the lack of studies using different ML algorithms on the same data has made it difficult to directly compare the results of the different algorithms.

Additionally, the investigation of brain anomalies in patients with mental disorders has been considerably hindered by the lack of precision in mapping the functional regions at the individual level (23). Multiple studies have consistently indicated that functional organization can vary significantly among individuals, particularly in the higher-order association cortices (23–25). Precise identification of the functional nodes in individuals is necessary to detect neuroimaging biomarkers for mental illnesses due to the significant inter-subject variability. We and others have shown that brain-behavior correlations would be stronger when brain networks are established using individual features rather than a group-level atlas (26–29). Therefore, it is necessary to further explore the application prospect of a novel, individualized functional network parcellation analysis in predicting treatment response.

Given such a background, we mapped the fine-grained functional regions in each subject and constructed individualized structural morphology and functional topological features to predict the one-year treatment response in schizophrenia. Additionally, we used the same analysis pipeline for different ML algorithms to directly compare the differences in model predictions between different algorithms. We trained ML models to predict the treatment response using the training and validation cohorts and assessed the model’s generalizability in the external test cohort. We hypothesize that 1) individualized parcellation methods can further improve the predictive ability of schizophrenic clinical outcome than group-level templates, and 2) the predictive model performance of different ML algorithms may be different.

## 2 Materials and methods

### 2.1 Participants

We enrolled 263 drug-naïve patients with first-episode schizophrenia from the West China Hospital Mental Health Center of Sichuan University and an additional 110 drug-naïve first-episode schizophrenia patients from the Fourth People’s Hospital of Chengdu for our research. Diagnosis criteria of schizophrenia met the Structured Clinical Interview for DSM-IV (SCID) (30) and confirmed by consensus between two psychiatrists. Exclusion criteria comprised: (1) Axis I psychiatric disorders other than schizophrenia; (2) significant systemic or neurological illness; (3) alcohol or drug abuse; (4) pregnancy; and (5) MRI contraindications, including cardiac pacemakers and other metallic implants.

At baseline, no patients had received prior antipsychotic or psychiatric medication, and all participants underwent 3T MR head scans prior to treatment. Clinical symptom severity was assessed using the Positive and Negative Syndrome Scale (PANSS) for each patient. 87 patients were excluded from the study due to incomplete or poor-quality images and failure to complete the full assessment. After the baseline MR scan and symptom assessment, all patients received treatment with second-generation antipsychotic medications, with drug selection and dosage determined by the attending psychiatrist. Daily dosages of antipsychotic drugs were converted into chlorpromazine equivalents (31). Only 72 patients completed the 1-year follow-up, with 4 of them excluded due to poor-quality images caused by motion artifacts.

A total of 68 right-handed drug-naïve first-episode schizophrenia patients were included in this study, with 51 subjects from the West China Hospital for model training and validation, and 17 subjects from the Fourth People’s Hospital of Chengdu for model testing. The severity of psychiatric symptoms was assessed using the PANSS both at baseline and the 1-year follow-up. The percentage reduction of PANSS at follow-up was calculated as follow:


$$\frac{{\text{PANSS}}_{\text{baseline}}-{\text{PANSS}}_{\text{follow-up}}}{{\text{PANSS}}_{\text{baseline}}-30}\times 100\%.$$

A 50% reduction served as the criterion for treatment response (32). Subsequently, the subjects from the training and validation cohorts were segregated into a response group (RG, n = 38) and a non-response group (NRG, n = 13). The test cohort consisted of RG (n = 12) and the NRG (n = 5). The study received approval from the Ethics Committee on Biomedical Research, West China Hospital of Sichuan University. All participants provided written informed consent.

### 2.2 Image acquisition

#### 2.2.1 Training and validation cohort

At baseline, participants underwent brain scans using a 3T MRI system (EXCITE; General Electric, Milwaukee, Wisconsin) with an 8-channel phased-array head coil. High resolution 3D-T1 weighted images (3D-T1WI) were obtained using a three-dimensional spoiled gradient-recalled sequence with the following parameters: repetition time (TR), 8.5 ms, echo time (TE), 3.4 ms, flip angle, 12˚, and field of view (FOV) = 240 mm × 240 mm. The acquisition matrix, composed of 256 readings of 128 phase encoding steps, resulted in 156 contiguous coronal slices with a slice thickness of 1 mm. The final matrix was automatically interpolated in-plane to achieve an in-plane resolution of 0.47 mm × 0.47 mm. Resting-state functional MRI (rs-fMRI) was acquired using a gradient-echo echo-planar imaging sequence with the following parameters: TR = 2000 ms, TE = 30 ms, flip angle = 90˚, slice thickness = 5 mm, matrix size = 64 × 64, FOV = 240 mm × 240 mm, and voxel size = 3.75 mm × 3.75 mm × 5 mm. Each brain volume consisted of 30 axial slices, and each functional run included 200 image volumes.

#### 2.2.2 Test cohort

A 3T SIEMENS TrioTim scanner was used, equipped with a 32-chanel head coil. High resolution 3D-T1WI were acquired using an SPGR sequence with the following parameters: TR = 2400 ms, TE = 2.0 ms, flip angle = 8°, FOV = 256 mm × 256 mm, 208 contiguous sagittal slices with a thickness of 0.8 mm, and an in-plane resolution of 0.8 mm × 0.8 mm. Rs-fMRI was obtained using an echo-planar imaging sequence with the following parameters: TR = 700 ms, TE = 37.8 ms, flip angle = 52°, slice thickness of 2.1 mm (no slice gap), matrix size = 100 × 84, FOV = 210 mm × 176 mm, and voxel size = 2.1 mm × 2.1 mm × 2.1 mm. The functional data consisted of 64 axial slices of 2.1 dummy volumes and 415 sequential image volumes, acquired over a total time of 633 seconds.

### 2.3 Imaging preprocessing

High-resolution 3D-T1WI images were subjected to analysis using the standard recon-all pipeline within FreeSurfer software (version 6.0, available at http://surfer.nmr.mgh.harvard.edu/). The image processing pipeline encompassed several steps (33, 34): visual inspection for motion artifacts, non-brain tissue removal, transformation to Talairach space, segmentation of subcortical gray/white matter (GM/WM), intensity normalization, tessellation of the GM/WM boundary, automated topology correction, and surface deformation. These steps served to segment cortical structures for use as a template in subsequent rs-fMRI registration.

The Computational Brain Imaging Group (CBIG) toolbox, available at https://github.com/ThomasYeoLab/CBIG, was employed for preprocessing rs-fMRI data (23, 35). This preprocessing encompassed various steps, including slice time correction, motion correction through Framewise displacement (FDrms) and voxel-wise differentiated signal variance (DVARS) calculations, removal of frames with FDrms > 0.2 or DVARS > 50, spatial distortion correction, nuisance regression, temporal interpolation of censored frames, bandpass filtering in the range of 0.009-0.08 Hz, projections to the standard surface (fsaverage 5), and smoothing with a 6mm kernel.

### 2.4 Individualized and group-level functional networks parcellation

We utilized the individual-specific cortical functional network parcellation method developed by Kong et al. (29) from the CBIG toolbox, which employs a multi-session hierarchical Bayesian model (MS-HBM) to estimate individual-specific cortical networks. The MS-HBM is designed to distinguish within-subject (intra-subject) from between-subject network variability through multiple layers. For each participant, the bilateral cerebral hemispheres were parcellated into 17 functional networks (35), encompassing the visual A network (VisCent), visual B network (VisPeri), somatomotor A network (SomMotA), somatomotor B network (SomMotB), dorsal attention A network (DorsAttnA), dorsal attention B network (DorsAttnB), salience A network (SalA), salience B network (SalB), limbic A network (LimbicA), limbic B network (LimbicB), control A network (ContA), control B network (ContB), control C network (ContC), default A network (DefaultA), default B network (DefaultB), default C network (DefaultC), temporal parietal network (TemPar). In brief, this method comprises three key steps: (1) generating profiles and initialization parameters, (2) estimating group priors, and (3) generating individual-level parcellations. Following these steps, 17 individual-level cerebral functional networks were created in each hemisphere for every participant. The results of individual-level parcellation for five subjects are illustrated in **Supplementary Figure S1**. These networks retain the same names as listed above, but differ in their morphology, indicating variations in distribution or anatomical location within the same network.

A group-level parcellation was performed, resulting in 17 functional networks in each hemisphere for each subject based on the Yeo atlas (35).

The signal intensities of all vertices within the individual-specific or the group-level networks were averaged to compute the mean time series of each network. Subsequently, two functional network matrices (34 × 34) were generated for each individual based on the individualized and group-level parcellations. This was achieved by calculating the 34 functional networks spanning both hemispheres and applying a z-transformation.

### 2.5 Gray matter volume calculation and graph theory analysis

GMV for each functional network was extracted using FreeSurfer software. Individual-specific and group-level parcellations served as atlases for each subject, respectively.

GTA was conducted using the DPABINet module within the Data Processing and Analysis for Resting-State Brain Imaging (DPABI, http://rfmri.org/dpabi) toolbox (36). Certain graph theoretic parameters required normalization, and during the normalization process, random network graph theoretic parameters were employed as reference for normalization. Specifically, 100 random networks with an equivalent number of edges and nodes to the calculated network were randomly generated using DPABINet. The average values of their graph-theoretic parameters were utilized as the reference for normalization. Additionally, to enhance the generalizability of the results, a range of sparsity values, spanning from 0.01 to 0.5 with an interval of 0.01, was employed in the calculation of graph theory parameters. A density range was also calculated, ranging from 0.01 and 0.34 with an interval of 0.01 (37). Global topological properties encompass measures such as (1) local efficiency, (2) global efficiency, (3) clustering coefficient, (4) characteristic shortest path length, (5) small-worldness. Regional topological properties were the degree centrality of each network (See **Supplementary Table S1** for the means of topological properties). The area under the curve (AUC) for each network metric was calculated to provide an overall value for the topological characterization of brain networks independent of any specific cost threshold.

### 2.6 Model construction and comparison

We used the FeAture Explorer software (FAE), version 0.5.5, implemented in Python, to construct 10 machine learning (ML) algorithms. These models include support vector machine (SVM), auto-encoder (AE), linear discriminant analysis (LDA), random forests (RF), logistic regression (LR), logistic regression via Lasso (LRLasso), ada-boost (AB), decision tree (DT), Gaussian process (GP), and naïve Bayes (NB). The software is available at https://github.com/salan668/FAE.

FAE provides a comprehensive pipeline encompassing the following key stages: (1) Data balancing, addressing the imbalance of data between RG and NRG, we applied the synthetic minority oversampling technique (SMOTE) to ensure balance in the training and validation cohorts. SMOTE achieves this by introducing synthetic examples along line segments, connecting them with the nearest k minority class neighbors, and choosing neighboring points randomly based on the required oversampling volume. (2) Normalization, we normalized the data using Z-score method. (3) Data preprocess, we employed the Pearson correlation coefficient (PCC) within FAE. This involved traversing all features, calculating the Pearson correlation coefficient pairwise, and randomly removing one of them when the coefficient exceeded the threshold of 0.99 to ensure that features did not exhibit excessive similarity. (4) Features selection, we considered feature numbers ranging from 1 to 15 (A simple “rule of thumb” for prognostic research requires a minimum of ten samples per feature) (38) and employed three feature selection methods: One-way analysis of variance (ANOVA) (with a threshold of 0.90), Recursive Feature Elimination (RFE), and Kruskal-Wallis test (KW); (5) Classification, classification performances were evaluated using ML algorithms implemented in Python with the scikit-learn library (https://scikit-learn.org/).

To mitigate the heterogeneity introduced by different institutions that could affect the comparison of features, we employed ANOVA to ascertain whether the GMV and GTA features of each sequence significantly differed among institutions. If a feature exhibited a significant difference, we harmonized it using the ComBat method (39).

**Figure 1:**
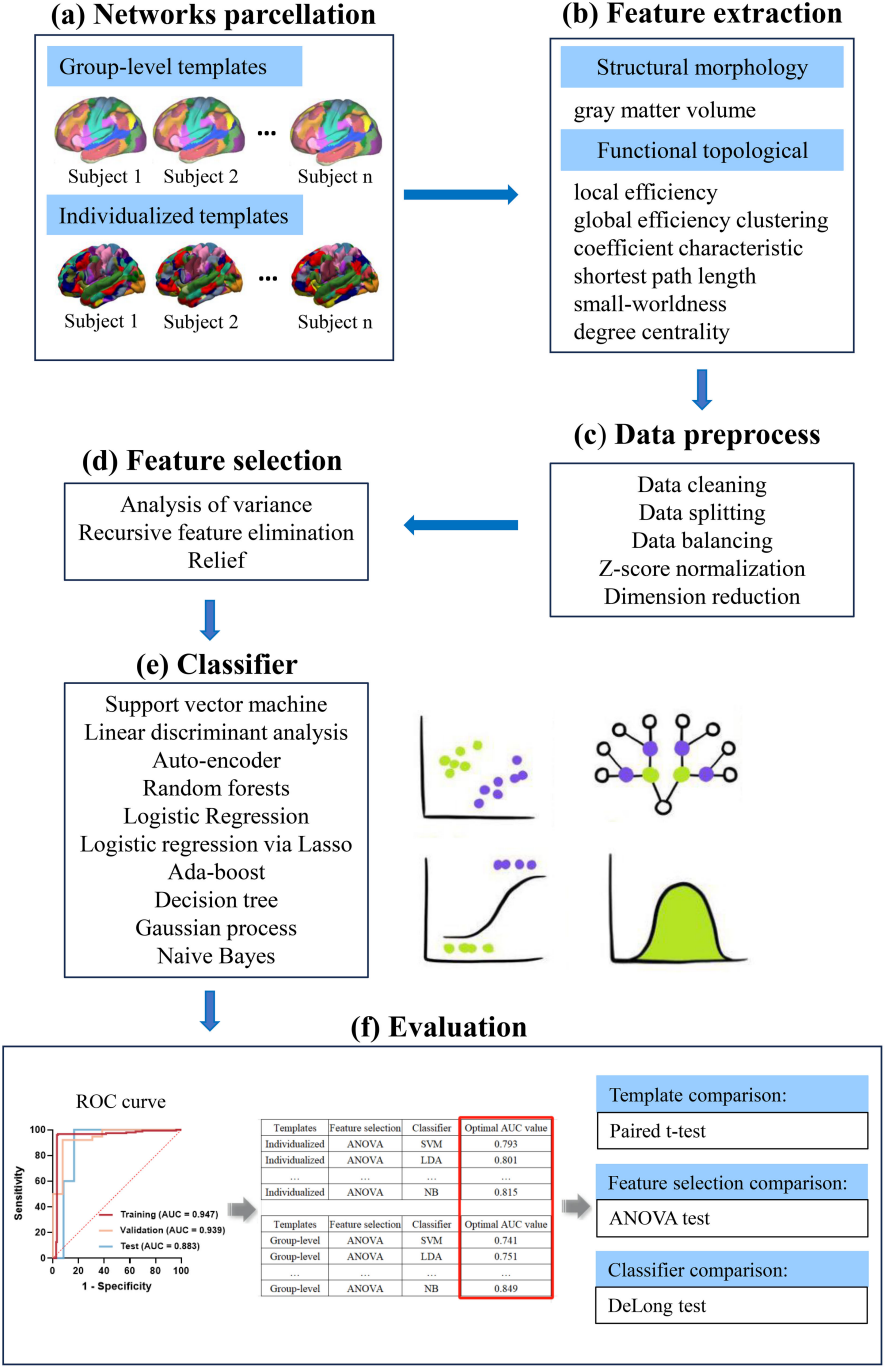
A schematic diagram for the whole machine learning pipeline. **(A)** Individual-specific parcellation were generated by employing a multi-session hierarchical bayesian method. Then, functional network matrices (34 × 34) were calculated for each subject based on the individualized and group-level templates. **(B)** Structural morphology- and functional topological-based features were extracted from functional network matrices. **(C)** Data preprocess. **(D, E)** Three feature selection methods and 10 classifiers were used to construct the models. **(F)** ROC curve analysis was employed to quantify the performance of the model in the training, validation and test cohort. The optimal AUCs of models constructed by different template parcellation techniques, feature selections and classifiers were compared. ANOVA, one-way analysis of variance; AUC, area under the curve; ROC, receiver operating characteristic.

Thus, the various methods mentioned above were combined, leading to the construction of a total of 450 ML models for features. These models were established based on individualized and group-level network parcellation, respectively (1 normalization × 3 feature selection × 15 feature number × 10 classifier). The results were assessed through a 5-fold cross-validation and validated using the test cohort. Evaluation metrics, including accuracy, sensitivity, specificity, negative predictive value (NPV), and positive predictive value (PPV), were computed at the optimal cutoff value determined by maximizing the Youden index. Additionally, the area under the receiver operator characteristics curve (AUC) was calculated for each tested condition. The machine learning workflow is depicted in **Figure 1**.

### 2.7 Statistical analyses

SPSS 25.0 software and R (version 4.2.1) were used to analyze the data in this study. Frequencies and percentages were used for categorical data and mean ± standard for data on continuous variables. We compared the optimal AUCs of models constructed by different template parcellation techniques, feature selections and classifiers (see **Supplementary Materials** for details, **Supplementary Figures S2–S4**). Specifically, paired t-tests were performed on the AUCs of individualized and group-level models constructed using ANOVA, RFE, and KW feature selection, respectively. ANOVA was conducted on the AUCs of the models constructed by the 3 feature selections in the individualized and group-level templates, respectively. Differences in AUC estimates between classifiers were compared using the DeLong test. Univariate Pearson correlation analysis was performed to evaluate the features selected for the individualized template in relation to the reduction in PANSS scores. The significance threshold was set at P < 0.05.

## 3 Results

### 3.1 Demographic and clinical data

**Table 1** displays the demographic and clinical characteristics of the participants. The education years of the RG were significantly higher than those of the NRG in training and validation cohort (P = 0.01). Both in the training and validation cohort and test cohort, there were no intergroup differences in age, sex, illness duration, or daily dosage of antipsychotics between the RG and NRG.

**Table 1:** Demographic and clinical characteristics of participant groups.

	Training and validation cohort			Test cohort		
	RG (n = 38)	NRG (n = 13)	*P* Value	RG (n = 12)	NRG (n = 5)	*P* Value
Age (years)	24.0 ± 7.2	26.7 ± 12.0	0.44	29.0 ± 10.3	32.4 ± 14.7	0.59
Male/Female	17/21	8/5	0.30	5/7	1/4	0.39
Education (years)	13.2 ± 2.6	10.9 ± 2.7	**0.01**	11.6 ±3.1	8.0 ± 4.1	0.07
Illness duration (months)	6.6 ± 9.2	12.2 ± 17.5	0.29	31.4 ± 62.7	8.1 ± 10.0	0.13
Baseline PANSS						
Positive	25.3 ± 6.7	22.0 ± 5.7	0.12	24.8 ± 4.0	25.8 ± 4.1	0.63
Negative	18.0 ± 6.7	20.9 ± 7.0	0.19	21.0 ± 4.2	21.4 ± 3.6	0.86
Total	91.4 ± 14.5	92.5 ± 15.9	0.81	93.8 ± 13.6	92.4 ± 13.4	0.85
Follow-up PANSS						
Positive	8.8 ± 3.4	16.5 ± 6.6	**0.001**	10.9 ± 2.8	20.4 ± 4.0	**< 0.001**
Negative	11.4 ± 4.5	19.0 ± 4.6	**< 0.001**	12.8 ± 3.2	16.0 ± 2.2	0.07
Total	43.0 ± 10.1	77.0 ± 23.2	**< 0.001**	50.3 ± 8.6	76.4 ± 4.0	**< 0.001**
Percentage PANSS reduction (%)	79.3 ± 14.2	25.7 ± 27.6	**< 0.001**	68.0 ± 11.6	23.0 ± 17.4	**< 0.001**
CPZ equivalents (mg/day)	261.8 ± 182.4	200.6 ± 128.8	0.24	304.3 ± 79.3	256.1 ± 125.5	0.30

Data are expressed as mean ± standard deviation, unless specified. The p values smaller than 0.05 were shown in bold front. RG, responder group; NRG, non-responder group; PANSS, Positive and Negative Syndrome Scale; CPZ, chlorpromazine.

### 3.2 Data balancing and heterogeneity testing

The SMOTE was employed to automatically generate 25 synthetic samples for the NRG, mitigating the impact of an imbalanced dataset on classifier fitting. A comparison of the AUC of all pipelines was conducted using the validation dataset with FAE. ANOVA showed no significant differences in features between the different institutions (P > 0.05).

### 3.3 Comparison of templates, feature selection methods and classifiers

**Figure 2:**
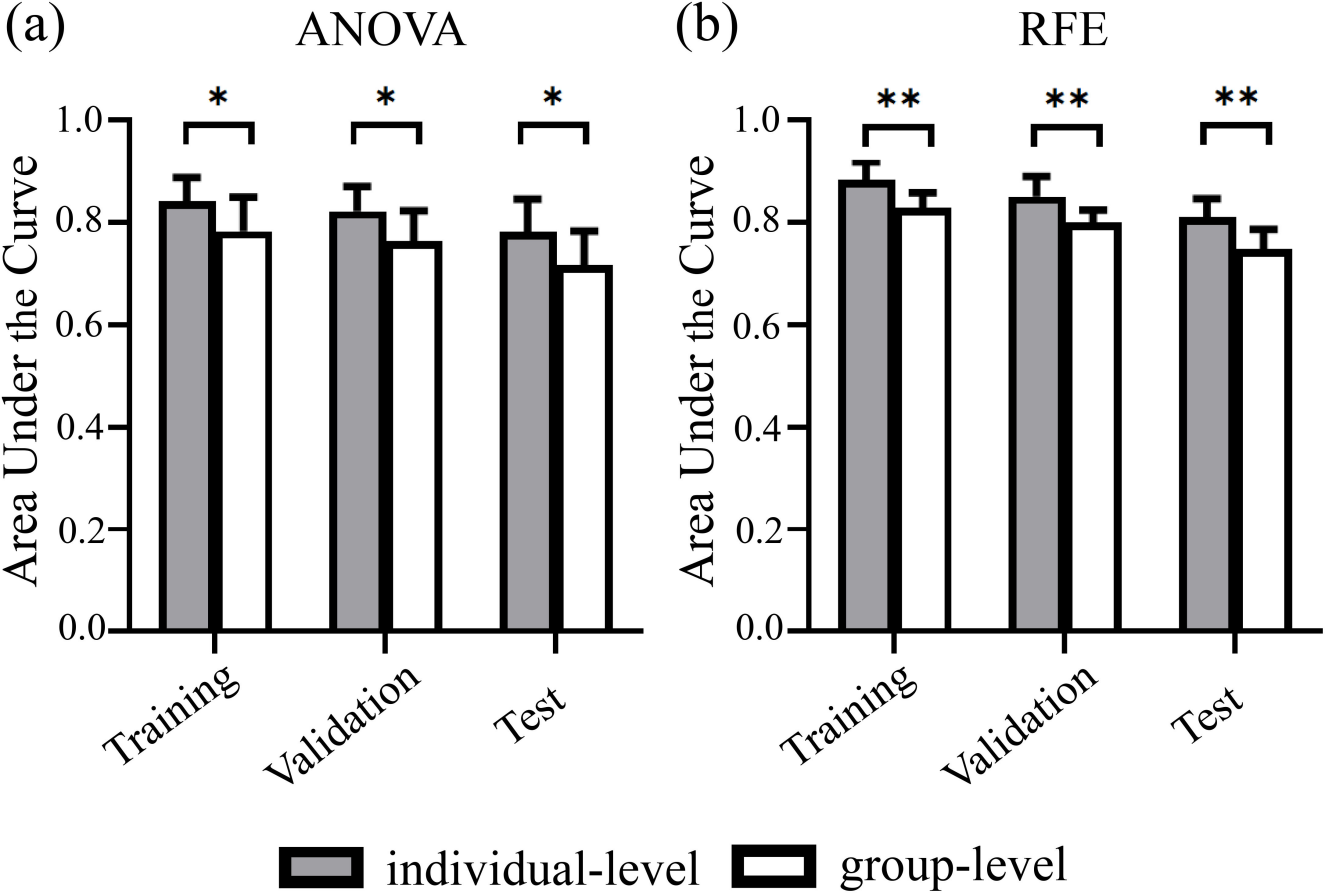
Predictive performance of individualized and group-level models at different datasets. Paired t-tests analysis results showed significant between-group differences in the AUCs of individualized and group-level models constructed using **(A)** ANOVA and **(B)** RFE feature selection, respectively. Error bars denote standard deviations. ANOVA, analysis of variance; RFE, recursive feature elimination. ^*^ indicates P <.05. ^**^ indicates P <.01.

**Figure 3:**
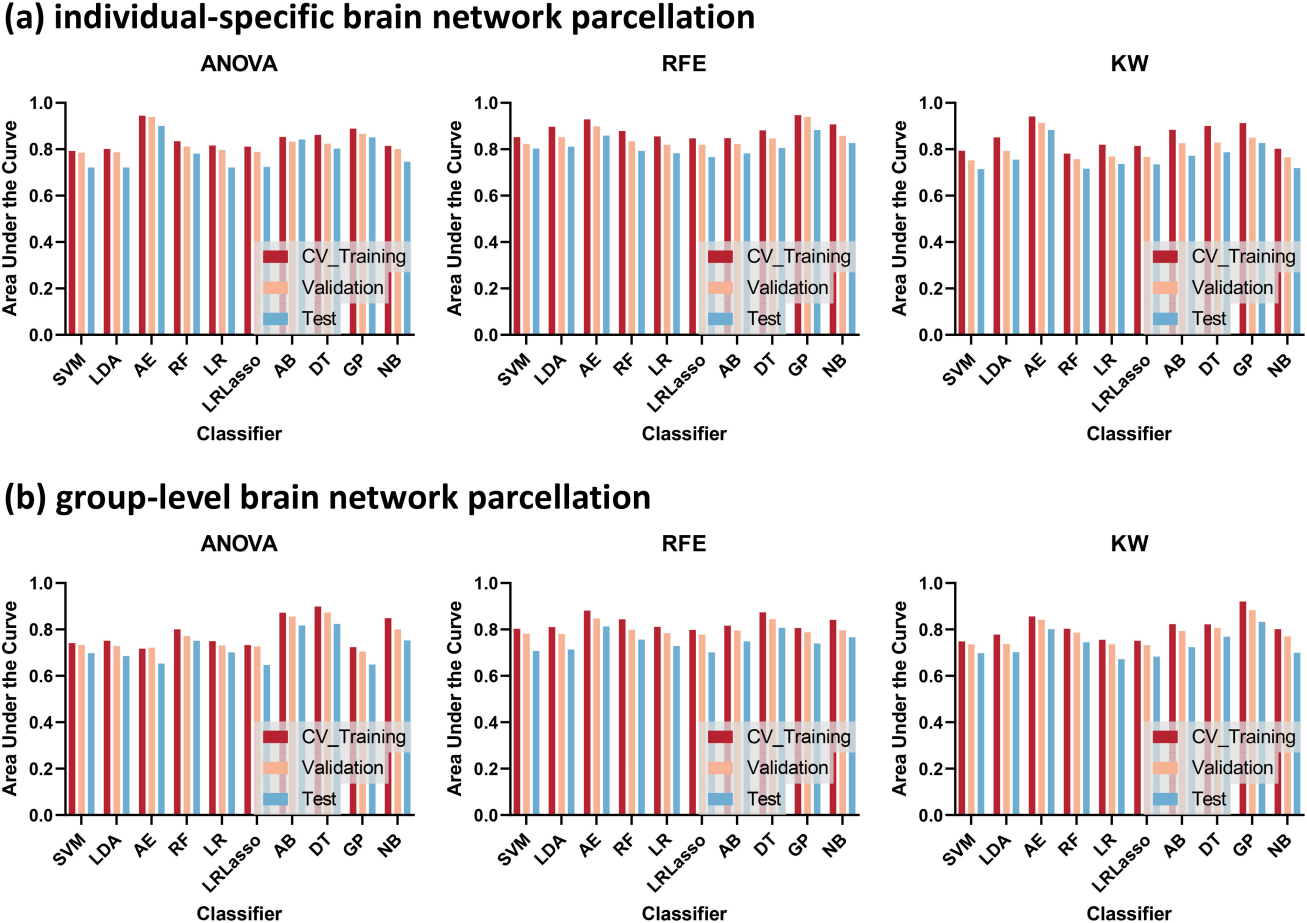
The optimal areas under the curve (AUCs) for 10 classifiers at different datasets. Template parcellation techniques using **(A)** individual-specific brain network, **(B)** group-level brain network. Feature selections using recursive feature elimination (RFE), analysis of variance (ANOVA), and kruskal-wallis test (KW).

Significant between-group differences in the AUCs of individualized and group-level models constructed by ANOVA and RFE, respectively (all P < 0.05) (**Figure 2**). However, the AUCs of the models constructed by the 3 feature selection methods and 10 ML algorithms showed no significant differences (all P > 0.05). **Figure 3** illustrates the optimal AUCs for 10 classifiers at different datasets.

### 3.4 Receiver operating characteristic curves and correlation scatter plots

**Figure 4:**
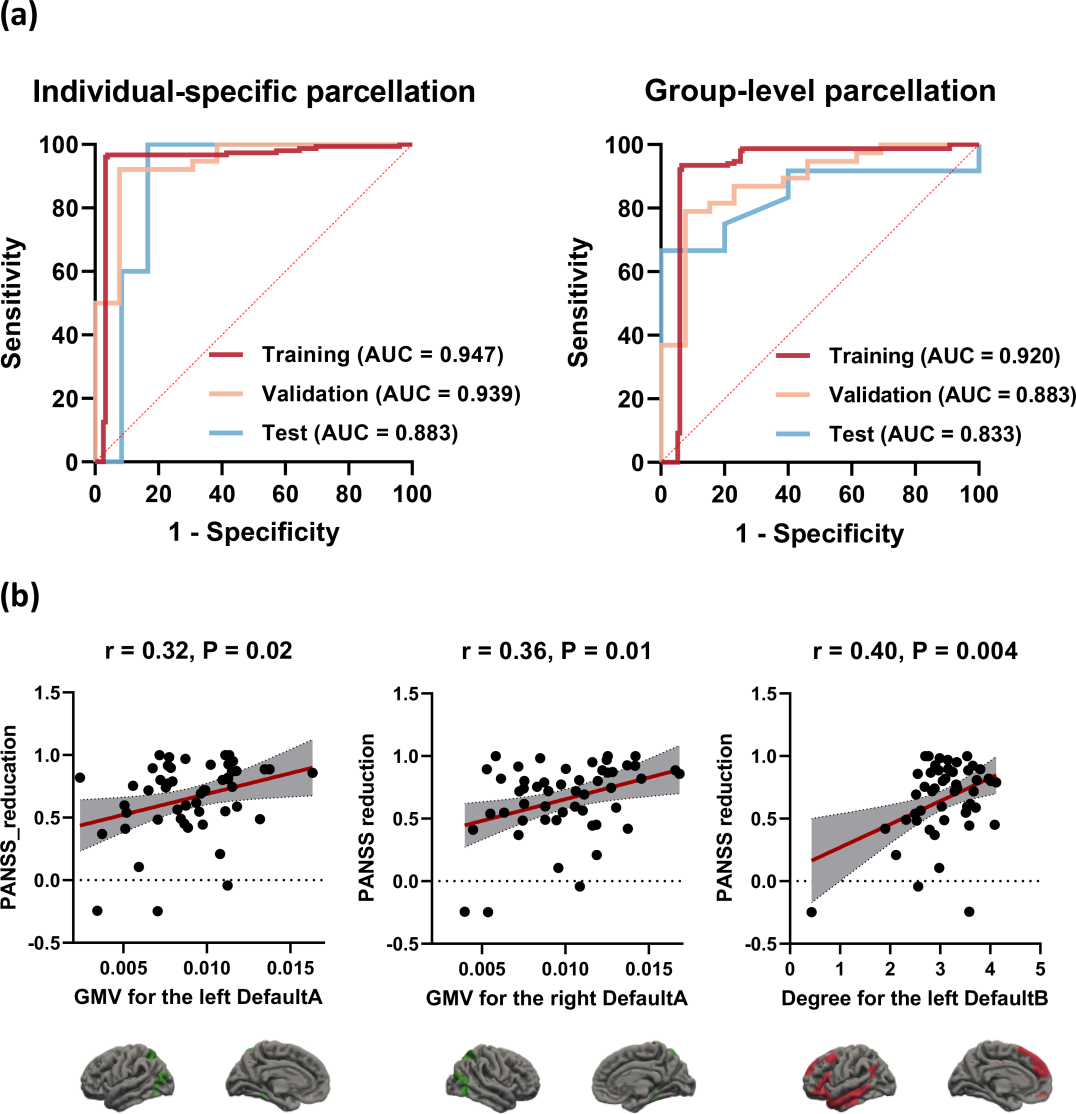
Receiver operating characteristic (ROC) curves and correlation scatter plots. **(A)** Optimal performance of the individual-specific and group-level model at different datasets. **(B)** The GMV for the bilateral DefaultA (r = 0.32/0.36, P = 0.02/0.01, for the left/right, respectively) and the degree for the left DefaultB (r = 0.40, P = 0.004) derived from the individualized template were significantly positively associated with PANSS reduction. DefaultA, default A network; DefaultB, default B network; GMV, gray matter volume.

As for individualized brain network segmentation templates, the pipeline employing the GP classifier achieved the highest AUC using 8 features with a “one-standard error” rule. The AUCs for the training, validation, and test cohorts were 0.947, 0.939, and 0.883, respectively (**Table 2**, **Figure 4A**). The selected features included the GMV of the bilateral DefaultA, the GMV of the left DefaultB and DefaultC, the degree of the bilateral ContA, the degree of the left LimbicA, and the degree of the left DefaultB. Feature selection was performed using RFE. These 8 features were all ranked first out of the 25 ranks derived by the RFE, which indicated the importance or contribution of each feature to the model’s performance, with lower ranks suggesting lesser importance.

**Table 2:** Optimal performance of the individual-specific and group-level model at different datasets.

	AUC	95% CIs	Acc	Sen	Spe	PPV	NPV
Individual-specific							
Training	0.947	0.914-0.980	0.965	0.967	0.962	0.961	0.968
Validation	0.939	0.860-0.999	0.923	0.921	0.929	0.972	0.813
Test	0.883	0.713-1.000	0.895	1.000	0.857	0.714	1.000
Group-level							
Training	0.920	0.880-0.961	0.938	0.941	0.934	0.935	0.940
Validation	0.883	0.776-0.989	0.823	0.789	0.923	0.968	0.600
Test	0.833	0.637-1.000	0.706	0.667	0.800	0.889	0.500

AUC, area under the receiver operator characteristics curve; CI, confidence interval; Acc, accuracy; Sen, sensitivity; Spe, specificity; PPV, positive predictive value; NPV, negative predictive value.

As for group-level brain network segmentation templates, the pipeline employing the GP classifier achieved the highest AUC using 14 features. The AUCs for the training, validation, and test cohorts were 0.920, 0.883, and 0.833, respectively (**Table 2**, **Figure 4A**). The selected features included the GMV of the left VisCent (F = 2.94), SalVentAttnA (F = 3.05), and ContC (F = 6.58), the GMV of the right SalVentAttnB (F = 2.76), LimbicA (F = 2.83), and DefaultA (F = 2.46), the degree of the bilateral SomMotB (F = 5.86/4.90 for left/right), the degree of the left DorsAttnB (F = 4.49) and DefaultB (F = 4.32), the degree of the right SomMotA (F = 4.85), LimbicA (F = 3.12), ContA (F = 9.08) and ContC (F = 3.16). Feature selection was performed using the KW. F-value is the statistical metric used in the KW test, a higher F-value indicates its potential importance.

Correlation analysis was conducted on the 8 features selected for the individualized template in relation to PANSS reduction. The features that demonstrated correlation with PANSS reduction were the GMV for the bilateral DefaultA (r = 0.32/0.36, P = 0.02/0.01, for the left/right, respectively) and the degree for the left DefaultB (r = 0.40, P = 0.004). **Figure 4B** shows the correlation scatter plot.

## 4 Discussion

This study explored the potential of the individual-specific network parcellation in predicting TRS through the application of various feature selection methods and state-of-art ML algorithms. Firstly, ML algorithms based on individual-specific brain networks demonstrated greater effectiveness in predicting outcomes than those based on group-level brain networks. The most predictive features for TRS based on individual-specific parcellation involved the GMV of the default network and the degrees of the control, limbic, and default networks. Secondly, although the pipeline employing RFE for feature selection and a GP classifier yielding the highest AUC, the prediction performance of models constructed by the three feature selection methods and ten ML algorithms showed no significant differences. In summary, our study highlighted the potential of individual-specific network parcellation in TRS prediction and underscored the crucial role of feature attributes in predictive model accuracy.

Prediction models utilizing individualized brain network parcellation showed superior classification accuracy than those based on group-level brain networks. The unique features of each individual likely derive from interindividual heterogeneity in the arrangement of brain functioning networks (24, 40–42). For example, variations in the shape, size, and position of functional areas may provide nonredundant information related to neurobehavioral abnormalities (28). Thus, consideration of individual variability in cortical anatomy will significantly preserve personal characteristics applicable to psychiatric applications (43). In contrast, functional regions based on a nominal “average” brain, potentially mis-localizing individuals’ functional regions and blurring biologically spatial signals (26, 44), particularly in the association networks that exhibit weak connections to anatomical structures (23–25). An instance of a function-anatomy dissociation that has been extensively investigated is the language network, wherein different subjects exhibit left-hemisphere or right-hemisphere dominance (45, 46). More generally, the inter-individual heterogeneity observed in association functions may represent a fundamental principle governing brain organization and an important outcome of the evolutionary trajectory of the human brain (47). Recognizing the importance of inter-subject variability in functional organization, neuroimaging community has been rapidly advancing in its efforts to map functional regions at the individual level (23, 25, 29). Wang and colleagues proposed an iterative parcellation procedure to localize the cortical functional networks in individual subjects and indicated that the outcomes were similar to the existing benchmark, invasive cortical stimulation mapping, in patients who were undergoing brain surgery (23). Kong et al. recently developed the MS-HBM parcellations that differentiates not only inter-subject network variability, but also within-subject network variability (29). Recent research found that crucial brain network characteristics may be missing in group-based templates but are apparent within individuals (25, 48). Using the group-level atlas on individual participants can weaken brain-behavior associations that are crucial for comprehending the particular illness processes (26–29). Our findings, together with those of the cited studies, collectively demonstrates that technological advances in subject-level functional mapping will not only enable the examination of functional dynamics within subjects, which are critical for personalized medicine, but will also contribute to traditional group-level studies by offering more significant indicators for comparing different subjects. Specifically, matching participants based on homologous functional regions will enhance the specificity of function signals in the networks under investigation, resulting in greater statistical power in group-level analyses.

The most predictive features for TRS based on individual-specific parcellation involved the GMV of the default network and the nodal degree of the control, limbic, and default networks. The default network is responsible for self-referential mental processes. Schizophrenia-related gray matter volume atrophy (49) and functional abnormalities during specific tasks (50) and rest (51, 52) in this region are regularly reported. Established disruptions in functional connectivity within the default network and between the default network and other task-relevant networks have been shown to be related to treatment response in schizophrenia (4, 53, 54). In fact, our univariate correlation analysis also revealed that increased GMV and nodal degree of the default network were associated with improved symptoms in schizophrenia. In contrast, the control network is involved in externally directed cognitive control functions and has been associated with working memory, attention, relational integration, and response inhibition (55–57), functions known to be impaired in schizophrenia. The limbic network dysfunctions may contribute to amplified threat processing and impaired emotion regulation (58–60). Taken together, the abnormalities in the control, limbic, and default networks may be related to disruptions in the balance between the internal stimuli, external perception, and emotional regulation, thereby contributing to the persistence of symptoms characteristic of TRS. Additionally, given the large inter-individual variability of associative cortices, such as default network and control network, our results suggest that it is critical to locate the boundaries of functional networks across individuals, as mislocating the networks will significantly obscure the true value of low-amplitude correlations between networks and hinder the discovery of markers of treatment response.

Although the pipeline employing RFE for feature selection and GP classifier yielded the highest AUC, the prediction performance of models constructed by the three feature selections and ten ML algorithms showed no significant differences. This is rather unexpected given that, despite recent evidence demonstrating that some applied classifiers share mathematical similarities (61), it is also evident that some of them are distinctly different. The most notable instance being the RF algorithm, which divides the feature space to binarize continuous variables, and is not restricted by the additivity found in LR and SVM algorithms. Each algorithm exhibits a preference for particular problem types over others and typically necessitates the adjustment of various configurations and parameters to achieve optimal performance on the dataset (62). However, Khondoker and colleagues reported the same findings in a classification study involving patients with Alzheimer and controls, showing that different classifiers tended to achieve similar levels of classification accuracy when effect size increased, diminishing the significance of algorithm selection (63). Similarly, two large-scale studies also suggested that the choice of ML algorithm for classification has less impact on final accuracy than the choice of measurement type (e.g., structural morphology, graph-based, and functional connectivity features) (16, 64). Perhaps, our results could be explained by a distribution of observations in the multidimensional feature space that mostly adheres to an unstructured pattern. A distribution with unstructured noise would not be more effectively classified by any complicated function than a hyperplane, which is a geometric feature that all classifiers can generate to a great extent (61). Consequently, this would ultimately result in similar classification accuracies. Additionally, some researchers (65) suggest that the difference in performance may be overshadowed by other uncertain data sources that are typically not taken into account in the traditional supervised classification framework (e.g., inappropriate assumptions and choices). Given the ongoing growth in computer technology, it is reasonable to expect that advancements will mostly stem from enhanced capabilities in data storage and processing. However, there are several factors that can determine the final accuracy of the model: 1) biological fingerprint, 2) sample size, 3) prediction algorithm, 4) data quality (64). The accumulating evidence and our findings have indicated that the biological fingerprint, as captured by the individualized imaging data in our study, is the most crucial factor influencing prediction performance.

Several limitations should be considered. First, drug selection and dosage were uncontrolled in this study. It is mostly due to the heterogeneity in antipsychotic medicines and the small sample size of our study. Controlling for these factors at 1-year follow-up is extremely challenging, and we frequently encountered loss to follow-up and refusals during this period, which may be a restriction for the study. Second, due to the study’s limited sample size, to include as many subjects as possible, there was a significant difference in the years of education, and the distribution of subjects in each group was not balanced. Although this variability may be attributed, at least partly, to differences in data availability and prevalence, we have addressed the issue of data imbalances as methodologically as possible. Although we hope that our findings could provide some initial insights into the clinical application role of individual-specific network parcellation and the value of feature attributes in predicting model accuracy outcomes in schizophrenic patients, we also consider that due to the small sample size these results to be rather pilot that need to be interpreted with caution. A deeper exploration employing larger samples and multicenter cohorts will be necessary. Third, at baseline, we did not collect whether patients had ever received psychotherapy and counseling intervention upfront, which people often use without a prescription to prevent or reduce symptoms. Future studies collecting patients’ psychotherapy histories data could help stratify patients and improve the reliability of study. Finally, given the lack of a reliable technique for mapping individual-specific subcortical regions, subcortical biomarkers were not incorporated into our prediction model.

## 5 Conclusions

In summary, our study has demonstrated that ML algorithms based on individual-specific brain networks are more effective in predicting outcomes than those based on group-level brain networks. Furthermore, the prediction performance of models constructed using different feature selection techniques and classifiers showed no significant differences. Our study highlighted the potential of individual-specific network parcellation in TRS prediction and underscored the crucial role of feature attributes in predictive model accuracy.

## Data Availability

The original contributions presented in the study are included in the article/**Supplementary Material**. Further inquiries can be directed to the corresponding authors.
