# Supplementary material for: Individualized multi-modal MRI biomarkers predict 1-year clinical outcome in first-episode drug-naïve schizophrenia patients
Source: Front Psychiatry. 2024 Sep 13;15:1448145. doi: 10.3389/fpsyt.2024.1448145 (PMC11427343; doi:10.3389/fpsyt.2024.1448145)
Supplement: Supplementary file 1 [file DataSheet1.docx]

**Individualized multi-modal MRI biomarkers predict 1-year clinical outcome in first-episode drug-naïve schizophrenia patients**

**Figure S1.** Multi-session hierarchical Bayesian model captures differences across subjects.

**Figure S2.** Comparisons between individualized and group-level templates.

**Figure S3.** Comparisons in ANOVA, RFE, and KW feature selection.

**Figure S4.** Comparisons of 10 classifiers.

**Table S1.** The means of topological properties of the cerebral functional network.

#
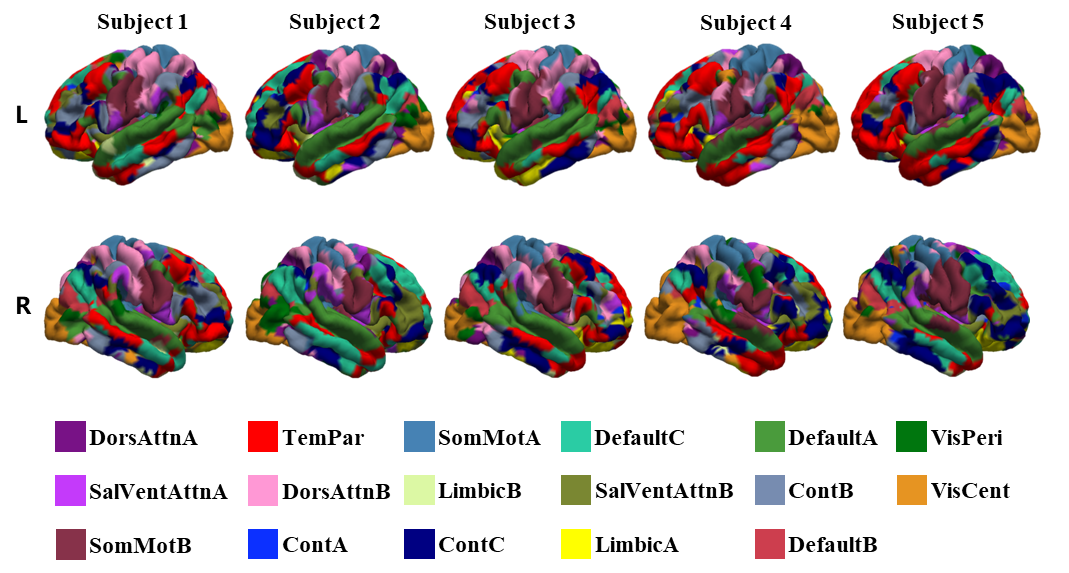
Figure S1. Multi-session hierarchical Bayesian model captures differences across subjects. The functional maps of the five randomly selected subjects varied substantially, especially in the higher-order association regions. Abbreviation: ContA, control A network; ContB, control B network; ContC, control C network; DefaultA, default A network; DefaultB, default B network; DefaultC, default C network; DorsAttnA, dorsal attention A network; DorsAttnB, dorsal attention B network; LimbicA, limbic A network; LimbicB, limbic B network; SalA, salience A network; SalAttnB; salience B network; SomMotA, somatomotor A network; SomMotB, somatomotor B network; TemPar, temporal parietal network; VisCent, visual A network; VisPeri, visual B network.

#
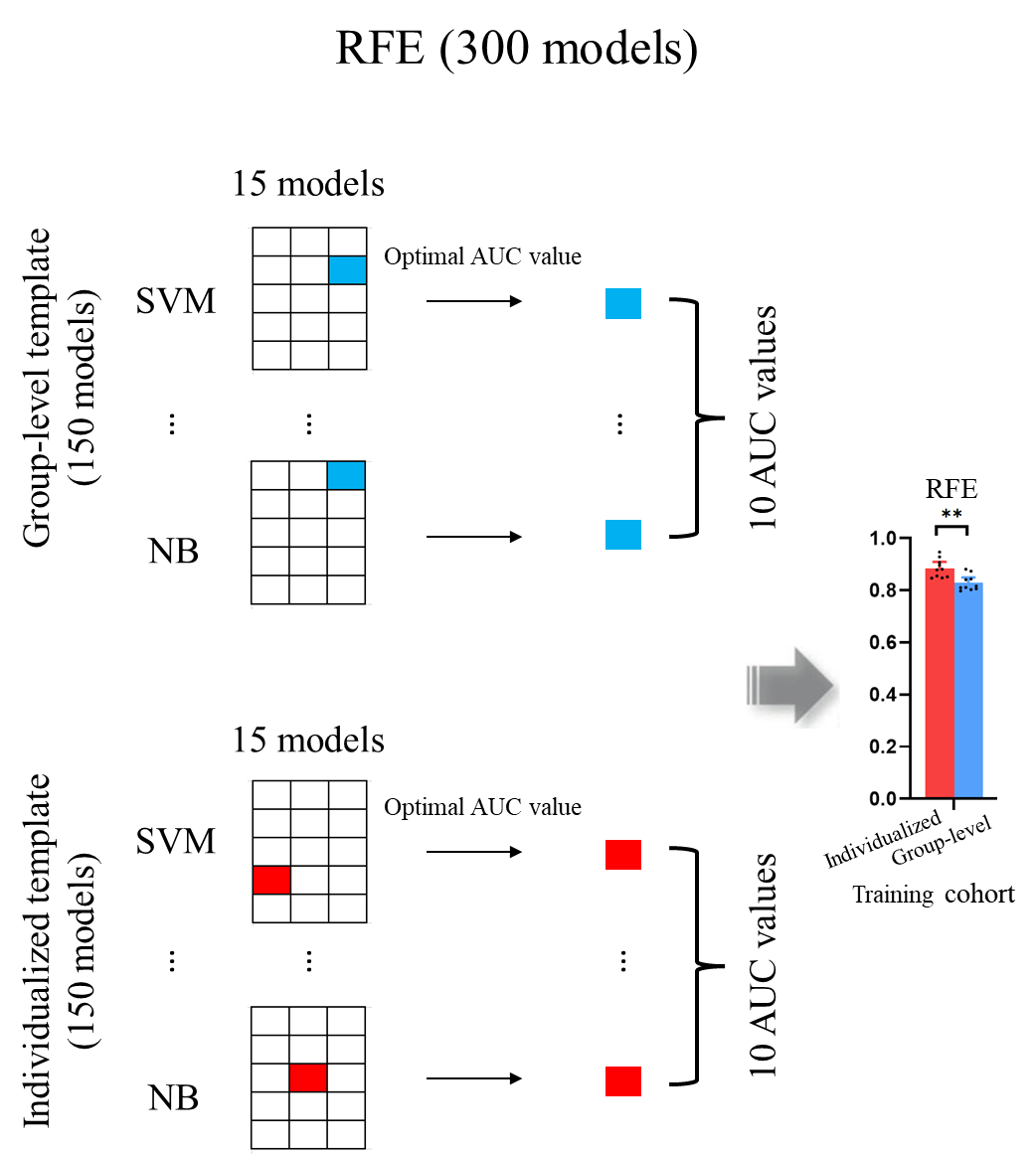
Figure S2. Comparisons between individualized and group-level templates. Paired t-test was performed on the AUCs of individualized and group-level models constructed using RFE feature selection in the training cohort. Abbreviation: AUC, area under the curve; NB, naive bayes; RFE, recursive feature elimination; SVM, support vector machine.


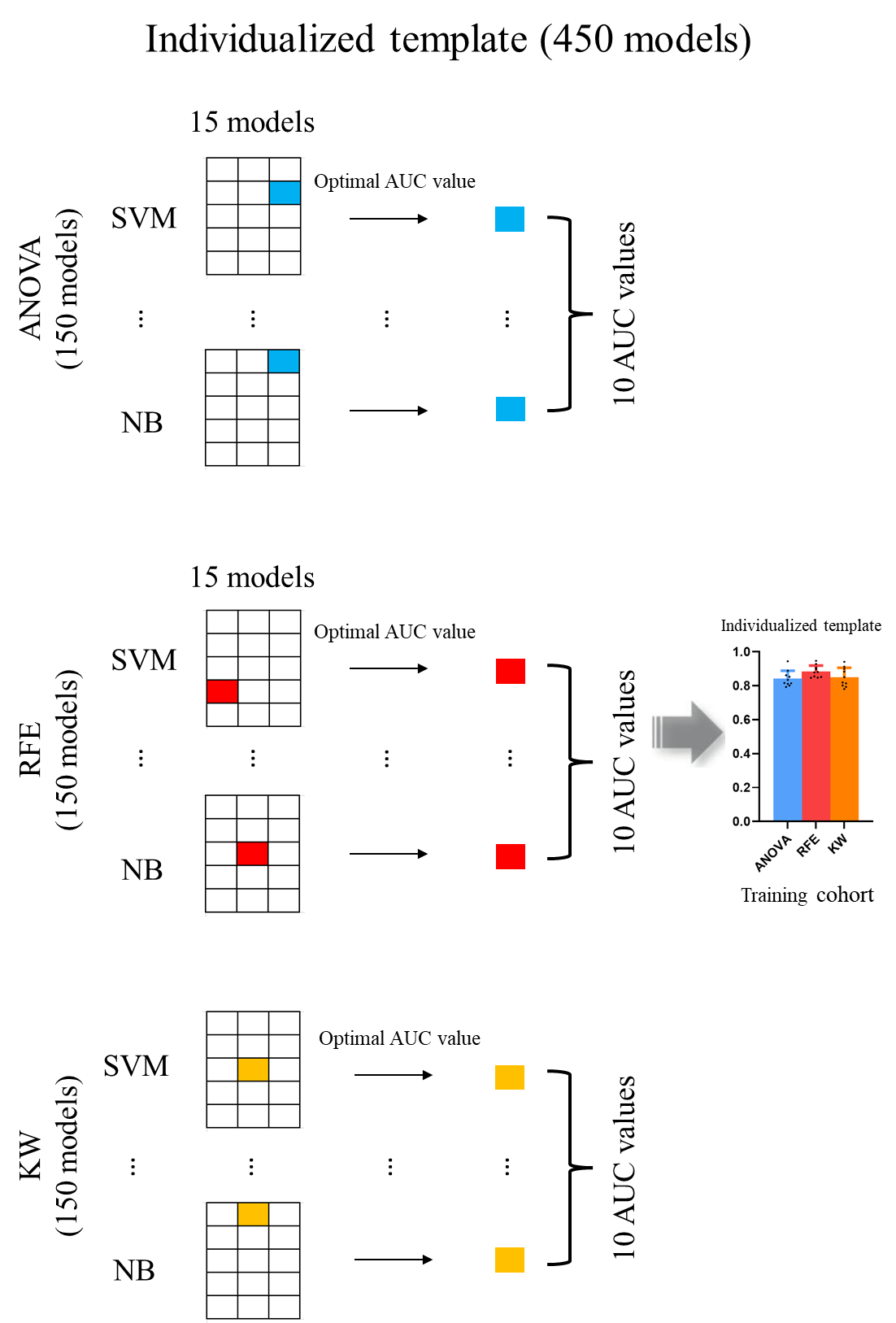
**Figure S3. Comparisons in ANOVA, RFE, and KW feature selection.** One-way analysis of variance tests was conducted on the AUCs of the models constructed by the 3 feature selections in the individualized templates. Abbreviation: ANOVA, one-way analysis of variance; AUC, area under the curve; KW, kruskal-wallis test; NB, naive bayes; RFE, recursive feature elimination; SVM, support vector machine.

**Figure S4. Comparisons of 10 classifiers.** DeLong test was performed on the AUCs of 10 classifier models constructed by individualized templates and RFE feature selection in the training cohort. Abbreviation: AUC, area under the curve; LDA, linear discriminant analysis; NB, naive bayes; RFE, recursive feature elimination; SVM, support vector machine.


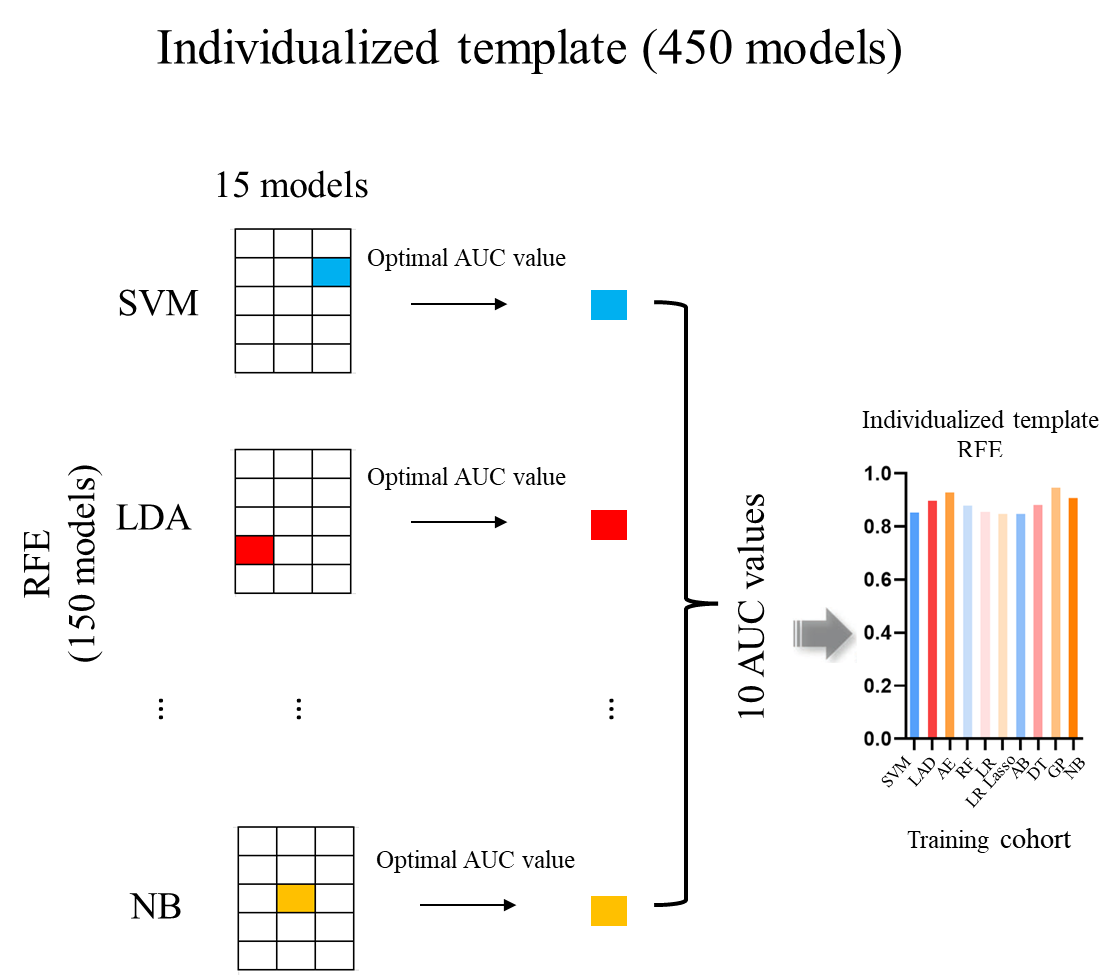


**Table S1. The means of topological properties of the cerebral functional network.**

| **Topological properties** | **Descriptions** |
| --- | --- |
| Global topological properties |  |
| Local efficiency | measures the capability of the network with regard to information transmission at the local levels |
| Global efficiency | measures the capacity of parallel information transfer over the entire network |
| Clustering coefficient | measures the ratio of existing connections among the neighbors |
| Characteristic path length | quantifies the mean of the shortest path length of any two nodes in the network |
| Regional topological properties |  |
| Degree centrality | reflects its impact on the flow of information between other nodes |
